# Editorial: Community series in interaction between the gut flora and immunity in intestinal diseases: volume II

**DOI:** 10.3389/fimmu.2026.1837636

**Published:** 2026-04-28

**Authors:** Yating Li, Silvia Turroni, Lan Gong, Ding Shi

**Affiliations:** 1 State Key Laboratory for Diagnosis and Treatment of Infectious Diseases, National Clinical Research Center for Infectious Diseases, Collaborative Innovation Center for Diagnosis and Treatment of Infectious Diseases, Department of Infectious Diseases, The First Affiliated Hospital, College of Medicine, Zhejiang University School of Medicine, Hangzhou, China; 2 Unit of Microbiome Science and Biotechnology, Department of Pharmacy and Biotechnology, University of Bologna, Bologna, Italy; 3 St. George & Sutherland Clinical School, Microbiome Research Centre, University of New South Wales, Sydney, NSW, Australia

**Keywords:** gut microbiota, immune regulation, intestinal diseases, microbiota-immune interactions, postbiotics

The interplay between gut microbiota and host immunity is now recognized as a key determinant of intestinal and whole-body homeostasis. Disruption of this delicate balance—often referred to as dysbiosis—has been linked not only to inflammatory bowel disease (IBD) and irritable bowel syndrome (IBS), but also to extra-intestinal conditions such as type 2 diabetes mellitus (T2DM) and chronic obstructive pulmonary disease (COPD). This Research Topic, the second volume of our Community Series, brings together seven articles that collectively advance our mechanistic understanding of microbiota-immune interactions and explore novel therapeutic strategies targeting this axis. The Research Topic includes foundational mechanistic reviews, disease-specific applications, and original research that translates mechanistic insights into therapeutic validation.

## Mechanistic foundations of gut microbiota-immune crosstalk

Two comprehensive reviews in this volume provide essential frameworks for understanding how gut microbes influence host immunity. Yu et al. systematically dissect the immunoregulatory roles of microbial metabolites—short-chain fatty acids (SCFAs), tryptophan derivatives, and bile acids—highlighting their ability to influence T cell differentiation, macrophage polarization, and key inflammatory signaling pathways including nuclear factor kappa-B (NF-κB), the NLR family pyrin domain containing 3 inflammasome (NLRP3), and the Janus kinase/signal transducer and activator of transcription (JAK-STAT) pathway. Complementing this, Wang et al. explore the reciprocal nature of host-microbiota dialogue, emphasizing how the immune system, particularly secretory immunoglobulin A (IgA) and T cell subsets, actively sculpts microbial community structure. Together, these reviews underscore that maintaining immune homeostasis requires a dynamic equilibrium, in which microbial metabolites serve as critical signaling molecules and the immune system acts as an ecological filter that enforces microbial stability.

## Beyond the gut: metabolic and respiratory connections

The gut-lung axis has gained substantial traction; Ni et al. extend this framework to COPD, delineating how gut dysbiosis contributes to pulmonary immune dysregulation through metabolite-mediated signaling and immune cell trafficking. Similarly, Niu et al. examine T2DM through the lens of the “gut microbiota-metabolite-barrier-inflammation” axis, proposing that acupuncture—a traditional Chinese medicine modality—exerts its therapeutic effects by reshaping microbial composition, enhancing SCFA production, and strengthening intestinal barrier integrity. These contributions illustrate the systemic reach of gut microbiota and open new avenues for cross-disease therapeutic strategies.

## *Akkermansia muciniphila*: from correlation to causality?

Among gut commensals, *Akkermansia muciniphila* has emerged as a particularly intriguing therapeutic candidate. Xu et al. provide a comprehensive review detailing its ability to modulate intestinal immune cells, epithelial barrier integrity, and stem cell function through multiple mechanisms, including Toll-like receptor 2 (TLR2)/NLR family pyrin domain containing 6 (NLRP6) signaling and metabolite-mediated pathways. Building directly upon this mechanistic framework, the original research by Jiang et al. in this volume provides compelling experimental evidence that both live *A. muciniphila* and its culture supernatant can alleviate spontaneous colitis in interleukin-10 (IL-10) knockout mice—a well-established model of chronic, microbiota-driven intestinal inflammation. Specifically, the authors demonstrate that treatment with *A. muciniphila* supernatant suppresses pro-inflammatory cytokines (tumor necrosis factor, IL-6, IL-17, interferon-gamma), enhances anti-inflammatory mediators (IL-4, transforming growth factor-beta, IL-22), and upregulates the tight-junction proteins zonula occludens-1 (ZO-1) and occludin, thereby reinforcing epithelial barrier function. Untargeted metabolomic profiling of the culture supernatant identified adenosine and tryptophan derivatives as candidate bioactive molecules, providing a mechanistic link between microbial metabolism and immune modulation. This work represents a critical step from descriptive association toward causal understanding, positioning *A. muciniphila*-derived postbiotics as a promising strategy for IBD management.

## Polysaccharides as dual-action therapeutics

Natural polysaccharides represent another promising class of therapeutics that can modulate the microbiota and reinforce the barrier function simultaneously. Yang et al. provide a comprehensive review of polysaccharide-based interventions in IBD, detailing how these complex carbohydrates promote beneficial bacteria, enhance SCFA production, and upregulate tight-junction proteins. Of note, they introduce the emerging role of polysaccharide-based nanocarriers—such as chitosan, alginate, and hyaluronic acid formulations—that enable pH/reactive oxygen species (ROS)-responsive drug delivery and improve colonic targeting. This integration of traditional bioactive compounds with modern nanotechnology exemplifies a forward-looking strategy for precision intervention.

## From association to causality: challenges and future directions

The articles in this volume underscore a critical transition in the field: the shift from describing associations between microbiota and disease to elucidating causal mechanisms and developing targeted interventions. The original research by Jiang et al. exemplifies this transition, as it moves beyond observational findings to demonstrate that specific microbial metabolites can confer protection in a genetically susceptible host. However, the challenges ahead remain substantial: methodological heterogeneity, confounding by antibiotics, corticosteroids and other host metadata, inter-individual variability, and the complexity of establishing causality in human populations. Nevertheless, the tools to address these challenges are rapidly evolving. Multi-omics integration, gnotobiotic models with humanized microbiota, and organ-on-chip platforms offer pathways to dissect mechanisms with greater precision. Moreover, the development of next-generation live biotherapeutics, including engineered probiotics, and nanocarrier-enabled metabolite delivery holds promise for translating mechanistic insights into clinically viable therapies.

In conclusion, this second volume of our Community Series captures the breadth and depth of current research on microbiota-immune interactions in intestinal and extra-intestinal diseases. The seven contributions—ranging from foundational mechanistic reviews to disease-specific applications and original experimental validation—not only advance our mechanistic understanding but also chart a course toward personalized, multi-target interventions that leverage the gut ecosystem as a therapeutic ally. We thank all authors for their insightful contributions and hope that this collection will inspire further research into the causal pathways linking gut flora, immunity, and disease, ultimately benefiting patients with intestinal and systemic disorders.

